# RNA-seq liver transcriptome analysis reveals an activated MHC-I pathway and an inhibited MHC-II pathway at the early stage of vaccine immunization in zebrafish

**DOI:** 10.1186/1471-2164-13-319

**Published:** 2012-07-17

**Authors:** Dahai Yang, Qin Liu, Minjun Yang, Haizhen Wu, Qiyao Wang, Jingfan Xiao, Yuanxing Zhang

**Affiliations:** 1 State Key Laboratory of Bioreactor Engineering, East China University of Science and Technology, 130 Meilong Road, Shanghai, 200237, China

## Abstract

**Background:**

Zebrafish (*Danio rerio*) is a prominent vertebrate model of human development and pathogenic disease and has recently been utilized to study teleost immune responses to infectious agents threatening the aquaculture industry. In this work, to clarify the host immune mechanisms underlying the protective effects of a putative vaccine and improve its immunogenicity in the future efforts, high-throughput RNA sequencing technology was used to investigate the immunization-related gene expression patterns of zebrafish immunized with *Edwardsiella tarda* live attenuated vaccine.

**Results:**

Average reads of 18.13 million and 14.27 million were obtained from livers of zebrafish immunized with phosphate buffered saline (mock) and *E. tarda* vaccine (WED), respectively. The reads were annotated with the Ensembl zebrafish database before differential expressed genes sequencing (DESeq) comparative analysis, which identified 4565 significantly differentially expressed genes (2186 up-regulated and 2379 down-regulated in WED; *p*<0.05). Among those, functional classifications were found in the Gene Ontology database for 3891 and in the Kyoto Encyclopedia of Genes and Genomes database for 3467. Several pathways involved in acute phase response, complement activation, immune/defense response, and antigen processing and presentation were remarkably affected at the early stage of WED immunization. Further qPCR analysis confirmed that the genes encoding the factors involved in major histocompatibility complex (MHC)-I processing pathway were up-regulated, while those involved in MHC-II pathway were down-regulated.

**Conclusion:**

These data provided insights into the molecular mechanisms underlying zebrafish immune response to WED immunization and might aid future studies to develop a highly immunogenic vaccine against gram-negative bacteria in teleosts.

## Background

Zebrafish (*Danio rerio*) is a well-established vertebrate model of human development and hematopoiesis [1]. However, as the aquaculture industry grows to meet the needs of an expanding global population, there has been a rapid increase in the research of teleost physiology and immunology. The zebrafish model system has proven to be a useful tool for studying infectious diseases that are natural threats to fish species of important human food sources, such as rock bream (*Oplegnathus fasciatus*) and turbot (*Psetta maxima*) [2]. Several studies of the zebrafish immune system have provided initial insights into host-microbe interactions with both pathogens and commensals [3,4], and the molecular processes mediating clearance of pathogenic infections [5-7]. Not surprisingly, many features of the zebrafish defense responses following pathogen invasion resembled those of other mammals, including humans [7-9].

The adult zebrafish has been used in a few studies to investigate new vaccines against specific pathogenic species. For example, Novoa *et al.*[10] studied the efficacy of a vaccine derived by reverse genetics against viral hemorrhagic septicemia virus in fish by using the zebrafish as a model system, and revealed that the vaccine was protective even at low temperatures. In addition, Cui *et al.*[11] used zebrafish to study an attenuated live *Mycobacterium marinum* vaccine aimed at reducing mycobacteriosis in freshwater and marine fish. Finally, Xiao *et al.*[12] developed an edwardsiellosis zebrafish model to screen attenuated live *Edwardsiella tarda* vaccine candidates in order to identify those most highly effective for subsequent development for industry use. However, so far, no work involves the immune-related pathways underlying the zebrafish response to vaccination.

In order to design a novel and effective vaccine, it is essential to gain a comprehensive understanding of the immune responses elicited in host upon vaccination. To date, most of the studies of the teleost immune system have focused on head kidney or/and spleen [13]. However, the vertebrate liver has recently been recognized as an essential immune organ [14-16], accommodating a variety of cell types [14,17], including those primarily involved in immune activities. Since the liver receives blood from both the systemic circulation and the intestine, it is exposed to a wide array of antigens. Therefore, its immune-related cellular components can manifest a broad range of immune reactions [14,18]. For example, the liver lymphocyte population includes both innate immune cells (such as the macrophages and natural killer cells) and adaptive immune cells (such as conventional T lymphocytes [14,19] that recognize and respond to antigenic peptides presented by the major histocompatibility complex (MHC)-I or -II [18,20]). As such, different infectious pathogens would be expected to induce distinctive profiles of immune responses in the liver [21], which might be manipulated to create specific and effective therapeutic strategies.

Several methods exist by which to determine the comprehensive transcriptomic profile of a pathogen-specific immune response, including microarray and quantitative real-time PCR [22,23]. However, the high-throughput RNA sequencing (RNA-seq) technology offers several advantages over the other profiling applications. Not only is RNA-seq independent on predefined probes, which facilitates the discovery of new transcript variants, but the sequence platform also produces low background noise, which allow for distinction between closely homologous genes and detection of weakly expressed transcripts [24]. In addition, concurrent advances in the bioinformatic algorithms used to analyze the RNA-seq data have allowed for better interpretation of the whole transcriptomic profile and provided further insights into complex molecular processes. The RNA-seq approach has already been successfully applied to several infectious disease models of zebrafish [25-27], including zebrafish embryo infected with *Salmonella*[26], and adult zebrafish and embryos infected by *Mycobacteria*[25,28]. In addition, other fish species infection models have been subjected to RNA-seq analysis, including large yellow croaker (*Larimichthys crocea*) infected by *Aeromonas hydrophila*[29] and Japanese seabass (*Lateolabrax japonicus*) infected by *Vibrio Harveyi*[30], but the overall immune-related transcription profiles have differed among species [25-30]. No reports exist in the literature of RNA-seq technology used to analyze the changes in an infected fish transcriptome profile induced upon vaccine treatment.

Edwardsiellosis, caused by the gram-negative *Edwardsiella tarda*, is currently one of the most economically disastrous infectious diseases affecting the global aquaculture industry [31]. *E. tarda* displays polymorphic phenotypes and has a broad range of hosts from aquatic invertebrates to higher vertebrates, including birds, reptiles, mammals, and even humans [31]. In developing a putative live attenuated vaccine against edwardsiellosis, Xiao *et al.*[12] constructed an *E. tarda* mutant (WED) with low residual virulence. Although the mutant was capable of inducing robust protection in zebrafish and turbot, the antibody titers detected in sera were relatively low. By thoroughly understanding the immune-mechanism of zebrafish induced by the putative live attenuated vaccine, a more immunogenic vaccine may be able to be generated. To this end, we performed a comparative gene transcription analysis of livers from mock-immunized and WED-immunized zebrafish using RNA-seq technology to investigate their differential transcriptsomic profile. Furthermore, 12 genes associated with MHC antigen processing were analyzed by qPCR and the results revealed an activated MHC-I pathway and an inhibited MHC-II pathway during the early stage of vaccine immunization. It was prompted that WED conferred a robust protection in zebrafish by eliciting an effective cell immunity via the MHC-I pathway.

## Results

### RNA-seq of liver transcriptome

To better understand the early stage immune response of zebrafish immunized with WED, six Solexa cDNA libraries were constructed from the livers of mock-immunized and WED-immunized zebrafish (Additional file 1). Biological replicates were pooled to make representative samples for deep sequencing analysis. Across the two groups of triplicate data, after normalization of the generated 95 bp PE raw reads, 15,683,828, 13,040,780 and 25,660,654 reads were obtained from C1-C3, and 16,306,312, 15,589,848 and 10,906,906 reads from V1-V3, respectively. To assess the quality of sequencing, the reads were mapped to the zebrafish reference genome. From the reads of each group, successful mapping occurred for 10,823,266 (C1), 9,584,828 (C2), 18,321,987 (C3), 12,209,418 (V1), 11,675,593 (V2) and 8,605,104 (V3) reads. However, 4,860,562, 3,455,952 and 7,338,667 unmapped reads were generated from C1-C3, while 4,096,894, 3,914,255 and 2,301,802 unmapped reads were found in V1-V3, respectively; we plan to conduct *de novo* analysis of these unmapped reads to generate a better reference of immune-relevant genes in zebrafish.

### Analysis of differential expression among WED- and mock-immunized zebrafish liver

**Figure 1:**
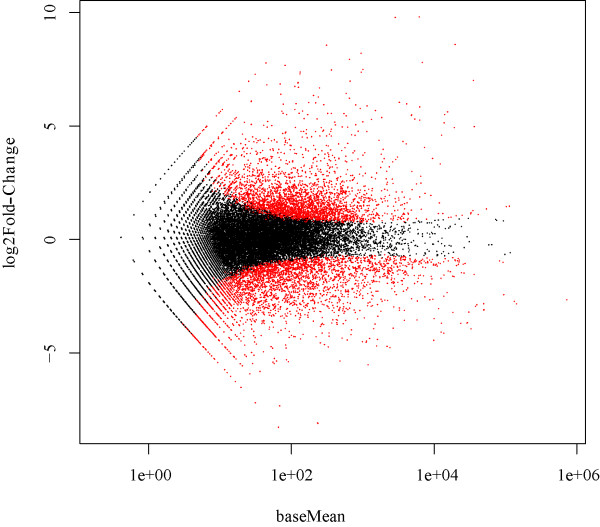
**MA plot of differentially expressed genes identified in WED-immunized and mock-immunized zebrafish livers.** Data represent individual gene responses plotted as log_2_ fold-change versus baseMean fold-change >2 (*p*-values <0.05), with a negative change representing the down-regulated genes and a positive change representing the up-regulated genes.

To identify the differentially expressed genes, the transcriptome data of zebrafish liver from two days after WED immunization and mock immunization were analyzed by using the DESeq package in R software [32]. The criteria of a two-fold or greater change in expression and *p*-value<0.05 (cut off at 5% FDR) were chosen to determine significantly up-regulated or down-regulated genes following immunization. The magnitude distribution of the significantly changed genes was illustrated by MA plot analysis (Figure 1). Using these criteria, a total of 4565 genes were significantly differentially expressed greater than two-fold, including 2186 up-regulated genes and 2379 down-regulated genes (Additional file 2). Annotation of the differentially expressed genes was achieved through BLASTN similarity searches against the Ensembl zebrafish RefSeq mRNA database (Version danRer 7, Additional file 2).

**Figure 2:**
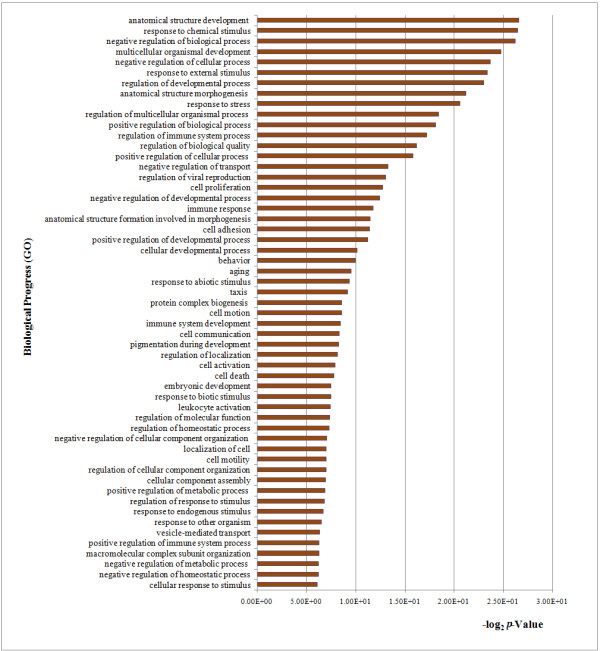
**Significantly up-regulated genes assigned to GO biological process categories**.

To perform an unbiased annotation of the functions of the differentially expressed genes identified by DESeq analysis, GO analysis of differentially expressed genes was carried out by two bioinformatics tools, DAVID and BiNGO plugin (Additional file 3). Among the 4565 differentially expressed genes, DAVID provided functional annotation for 3891 genes. GO annotated differentially expressed genes mainly belonged to the three functional clusters (biological process, cellular component, and molecular function), and were distributed among more than 70 categories (Additional file 3). The differentially expressed genes in the cluster of biological processes were found to be mainly related to stimulus response, immune response, regulation of immune system process, and regulation of development process (Figure 2).

To identify the biological pathways that are active in the zebrafish at the early stages of WED immunization, 4565 differentially expressed genes were mapped to canonical signaling pathways found in KEGG. A total of 3467 genes were mapped to 14 statistically significant categories (*p*<0.05; Table 1). Protein processing in ER was represented by 73 up-regulated and 8 down-regulated genes. There were also a statistically significant amount of mapped genes for other major antigen processing-related pathways, such as those mediated by “proteasome” and “protein export” pathway, to indicate the vital role of antigen processing and presentation activated by WED immunization at the early stage in zebrafish liver, which would elicit the specific immune responses required in the restoration of homeostasis.

**Table 1:** Statistically significant KEGG classifications of differentially expressed genes

**KEGG subcategories**	**Counts**	%*	***p*-value**
Ribosome	70	1.53	9.60E-14
Proteasome	35	0.77	1.80E-13
Protein processing in endoplasmic reticulum	81	1.77	2.27E-10
Arginine and proline metabolism	27	0.59	2.26E-04
Primary bile acid biosynthesis	10	0.22	8.23E-04
Fructose and mannose metabolism	18	0.39	2.44E-03
Glycolysis/Gluconeogenesis	27	0.59	3.47E-03
N-Glycan biosynthesis	22	0.48	3.71E-03
Glycine, serine and threonine metabolism	15	0.33	1.64E-02
Galactose metabolism	13	0.28	2.23E-02
Histidine metabolism	12	0.26	4.02E-02
Peroxisome	27	0.59	4.50E-02
Propanoate metabolism	13	0.28	4.84E-02
Protein export	13	0.28	4.96E-02

* indicates the percentage of genes in each pathway from 3467 genes mapped to KEGG.

In general, based on the results from GO analysis (by BiNGO plugin) and KEGG pathway analysis, the up- and down-regulated genes that were highly related to immune response of fish after WED immunization, significantly grouped into acute phase response (APR), complement activation, immune/defense response, and antigen processing and presentation pathway.

### The acute phase response is conserved in zebrafish liver following WED immunization

Most of the conserved acute phase response genes were significantly differentially expressed following WED immunization (Table 2). This set of genes encoded the major APPs (e.g. serum amyloid A (SAA), C-reactive protein (CRP), and serum amyloid P (SAP)), the minor and intermediate APPs (e.g. fibrinogen, plasminogen, plasminogen activator inhibitor, antitrypsin, ceruloplasmin, hemopexin, haptoglobin, and ferritin), the negative APPs (apolipoprotein A-IV and alpha-2-HS-glycoprotein), several complement components, and ion-binding and transporting proteins. Many of the APPs were up- or down-regulated greater than five-fold, suggesting that induction of APPs in zebrafish liver likely plays an important role in host defense stimulated by WED vaccine. Similar subcategories of APPs were also found to be differentially expressed in previous microarray-based studies of early stage immune response to bacterial infection in rainbow trout [33] and catfish [34], indicating the conservation of the vast majority of APPs among teleost fish. The major APPs, two CRP-like proteins (CRP and SAP) and SAA, were induced to up-regulated their expressions by 9.7-fold, 2.1-fold and 883-fold, respectively, in the WED-immunized zebrafish liver, emphasizing their importance in teleost innate immune response. In WED-immunized zebrafish, both the apolipoprotein A-IV, a fatty acid binding protein involved in extracellular and intracellular lipid transporting, and alpha-2-HS-glycoprotein were decreased by 3.0-fold and 11.8-fold, respectively. The decrease of apolipoprotein A-IV was consistent to previous report in starved zebrafish liver [35]. However, the functions of these two negative APPs in immune response remain unknown.

**Table 2:** Significantly differentially expressed genes in acute phase response

**Gene name**	**Accession number**	**Description**	**RPKM-C**	**RPKM-V**	**Fold-change**	***p*-value**
*ahsg*	ENSDARG00000069293	Alpha-2-HS-glycoprotein	554.80	55.81	−11.84	7.26E-50
*apoa4*	ENSDARG00000040298	Apolipoprotein A-IV	266.33	164.60	−2.09	2.21E-04
*tfrc*	ENSDARG00000012552	Transferrin receptor (p90, CD71)	23.29	13.29	−2.27	1.18E-04
*c1qb*	ENSDARG00000044612	Complement component 1, q subcomponent	6.06	3.14	−2.23	6.06E-03
*plg*	ENSDARG00000023111	Plasminogen	64.66	37.99	−2.18	6.73 E-04
*aerping1*	ENSDARG00000058053	C1 inhibitor	15.69	6.71	−2.93	5.49E-05
*c2*	ENSDARG00000019772	Complement component 2	24.57	52.88	2.06	2.99E-10
*si:dkey-8 k3.2*	ENSDARG00000038424	Complement C4	323.79	978.12	2.07	8.93E-13
*cfb*	ENSDARG00000055278	Complement factor B	727.97	1914.46	2.09	3.38 E-03
*apcs*	ENSDARG00000045089	Serum amyloid P component	309.74	872.88	2.16	1.64 E-04
*cp*	ENSDARG00000010312	Ceruloplasmin (Fragment)	633.50	1737.21	2.19	2.74 E-03
*c9*	ENSDARG00000016319	Complement component C9	18.78	48.27	2.28	4.90E-157
*cfhl2*	ENSDARG00000056778	Complement factor H, like 2	11.76	38.65	2.28	6.44E-07
*tfr2*	ENSDARG00000089980	Transferrin receptor 2	10.84	34.09	2.51	2.58 E-04
*fga*	ENSDARG00000020741	Fibrinogen alpha chain	375.41	1118.34	2.55	4.87E-05
*c3a*	ENSDARG00000012694	Complement component c3a	1305.16	4584.02	2.77	1.21 E-03
*serpina7*	ENSDARG00000087143	Antitrypsin	466.41	1720.58	3.05	1.18E-05
*c3*	ENSDARG00000043719	PREDICTED: complement C3-H2-like	154.97	692.50	3.44	1.19E-08
*serpine1*	ENSDARG00000056795	Plasminogen activator inhibitor type 1	13.14	68.84	4.38	6.20E-14
*cebpb*	ENSDARG00000042725	CCAAT/enhancer binding protein, beta	37.33	202.58	4.42	4.58E-13
*fgg*	ENSDARG00000037281	Fibrinogen, gamma polypeptide	154.90	965.71	5.39	6.08E-15
*fth1a*	ENSDARG00000015551	Ferritin	180.30	1233.87	5.45	9.51E-14
*f2r*	ENSDARG00000060012	Coagulation factor II (Thrombin) receptor	0.38	3.11	6.57	6.63E-05
*si:ch211-234p6.7*	ENSDARG00000071456	C-reactive protein	0.37	3.41	9.71	2.46 E-04
*atp7a*	ENSDARG00000003699	ATPase, Cu++ transporting	0.21	3.99	13.09	2.38E-06
*hpx*	ENSDARG00000051912	Hemopexin	192.84	7565.38	31.36	3.57E-17
*tlr5b*	ENSDARG00000052322	Toll-like receptor 5	2.17	98.99	36.41	7.93E-62
*lygl1*	ENSDARG00000056874	Lysozyme	11.85	641.71	41.37	1.70E-41
*c7*	ENSDARG00000057121	Complement component 7	6.38	435.85	63.02	4.13E-36
*itln3*	ENSDARG00000003523	Intelectin 3	52.98	8467.17	129.02	3.93E-33
*lect2l*	ENSDARG00000033227	Leukocyte cell-derived chemotaxin 2	8.75	2597.52	222.85	8.92E-56
*hp*	ENSDARG00000051890	Haptoglobin	14.36	7332.05	387.28	1.97E-30
*saa*	ENSDARG00000045999	Serum amyloid protein A	1.24	1248.94	883.07	2.56E-66

Identification of all differentially expressed genes was based on *p* < 0.05. A *p*-value < 0.05 indicated that the gene was significantly altered in WED immunized fish with respect to mock-immunized fish. The absolute value of “Fold-change” is the magnitude of up- or down-regulation for each gene/homolog after WED immunization. Fold-change > 2 indicates up-regulation, and < −2 indicates down-regulation.

Traditionally, complement has been considered as a supportive first line of defense against microbial intruders [36]. In WED-immunized zebrafish liver, three isoforms of complement C3, and the complement C4, C2, C7 and C9 were remarkably up-regulated. The C1 inhibitor and the C1q were up-regulated and down-regulated, respectively (Table 2).

Hepatocytes, which account for 80% of the liver mass [21], are the primary site of synthesis for all the genes involved in ion-binding and transporting [33,35,37]. In the RNA-seq data, more than 20 differentially expressed genes involved in ion-binding and transporting were strongly induced in zebrafish liver upon WED immunization (Table 2). These included haptoglobin, hemopexin, ceruloplasmin, transferrin receptor 2, ATPase, and Cu^2+^ transporting alpha polypeptide. Intelectin [33], which is involved in iron homeostasis, binding and transport, was one of the most up-regulated genes (129-fold) in the ion-binding and transporting category; However, the functions of intelectin in the contexts of normal iron metabolism and disease defense in zebrafish need to be further clarified. Members of the transferrin and ferritin families were significantly affected to result in obvious up- and down-regulation in zebrafish liver by WED immunization (Table 2). Leukocyte cell–derived chemotaxin 2 (LECT2) [35], originally named for its possible neutrophil chemotactic activity *in vitro*, was strongly induced by 222.8-fold in WED-immunized group, but its function in zebrafish remains unknown.

### WED immunization induces defense responses and signaling transduction pathways

**Figure 3:**
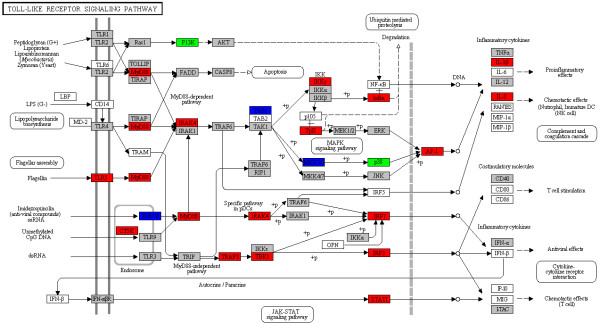
**Significantly differentially expressed genes identified by KEGG as involved in toll-like receptor signaling.** Red: significantly increased expression (fold-change >2); Blue: significantly decreased expression (fold-change <0.5); Green: genes detected both in up- and down-regulated groups; Gray: unchanged expression.

Functional annotation of significantly differentially expressed genes in zebrafish liver was performed to define the transcriptome profile more precisely. GO classification (Figure 2 and Additional file 3) indicated that immune/defense response-related genes were enriched, specifically under GO terms “response to chemical stimulus”, “regulation of immune system process”, and “immune response”. Toll-like receptors (TLRs) detected the presence of pathogens and triggered an innate immune response, and several of the differentially expressed genes from WED-immunized liver mapped to the TLR signaling pathways (Figure 3). TLR signaling has been remarkably conserved throughout evolution, and it can mediate immune responses to all types of pathogens and promote secondary disease. In zebrafish, the pathogenesis of *M. marinumStaphylococcus aureus*, and *Aeromonas salmonicida* has been shown to involve TLR signaling [25,35]. To further investigate the function of TLR5 a/b that elicited the immune response in zebrafish embryo, assessment of *tlr5a* and *tlr5b* by morpholino-mediated knockdown followed by flagellin stimulation clearly demonstrated TLR5-dependent gene activation of *mmp9, cxcl-C1c,* and *irak3*, which suggested that the activation of TLR5 pathway can induce the expression of inflammatory mediators as well as the feedback control of the innate immune response [38]. The functional investigation of TLR4 was also performed in a zebrafish embryo model, which suggested that the zebrafish TLR4 orthologs would negatively regulate the MyD88-dependent NF-κB activation by sequestering the TLR adaptors and indicated that the existence of a TLR would negatively regulate TLR signaling upon engagement with its specific ligand [39]. In humans and rodents, TLR-mediated signals in liver are associated with infection-induced granulomatous inflammation and ischemia-reperfusion injury, and can mediate liver regeneration processes [40]. In WED-immunized zebrafish liver, 21 differentially expressed genes mapped to various TLR pathways (Figure 3), including the TLR5, TLR8, TLR18 and TLR21 subcategories, which are not only expressed on the outer membrane of immune cells but also on endosome-lysosome membranes. TLR5 expression was up-regulated by 36.4-fold, indicating that it played a key role during the early stage of WED vaccination.

Immunity is a complex process of tightly controlled signals that involve a broad array of receptors, cytokines, enzymes, signal transducers, transcription factors, and other functional proteins. In our study, WED immunization increased dramatically the expression of cytokine genes related to the Jak-STAT, MAPK, TGF-β, apoptosis and VEGF signaling pathways. Therefore, WED-induced gene expression in zebrafish liver might facilitate protection against *E. tarda* by activating these pathways. Similar results were obtained in a previous study of large yellow croaker spleen during *A. hydrophila* infection [29]. Since the majority of the differentially expressed genes in these signaling pathways were up-regulated in our study, it is possible that the WED immunization of zebrafish is capable of triggering a vigorous adaptive immune response.

### WED immunization induces the antigen processing and presentation pathway

A large number of differentially expressed genes with functions in protein transportation, modification and degradation were up- or down-regulated in the zebrafish liver following WED immunization (Table 3), indicating these genes were likely connected to the degradation and processing of antigens for MHC class I and II molecules. Most of the differentially expressed genes related to MHC-I antigen processing pathways were significantly up-regulated, including the ER-resident chaperone calreticulin, calnexin, endoplasmin (grp94), TAP binding protein, proteasome activator (PA28), the heat shock proteins superfamilies, and cathepsin L. Meanwhile, the typical MHC-II processing pathway component, lysosomal membrane glycoprotein 2 (lamp2), was down-regulated (by 4.1-fold) in zebrafish liver after live attenuated vaccine immunization.

**Table 3:** Differentially expressed genes related to the antigen processing and presentation pathways

**Gene name**	**Accession number**	**Description**	**RPKM-C**	**RPKM-V**	**Fold-change**	***p*-value**
*ciita*	ENSDARG00000090851	MHC class II, transactivator	0.70	0.03	−26.80	5.54E-07
*si:busm1-160c18.3*	ENSDARG00000093706	Beta chain, MHC class II	2.04	0.36	−7.22	3.43E-04
*mhc2dab*	ENSDARG00000079105	MHC class II DAB gene	15.90	2.62	−7.22	2.16E-17
*si:busm1-194e12.12*	ENSDARG00000055447	Novel MHC II beta chain protein	6.12	1.70	−4.33	1.70E-06
*lamp2*	ENSDARG00000014914	Lysosomal membrane glycoprotein 2	3.47	1.11	−4.14	5.36E-03
*cd74*	ENSDARG00000036628	MHC, class II invariant chain, CD74 molecule,	34.87	10.77	−3.93	6.53E-12
*ctsz*	ENSDARG00000043081	Cathepsin Z	4.67	1.51	−3.77	8.36E-03
*ctssb.1*	ENSDARG00000074656	Cathepsin S, b.1	23.25	10.44	−2.59	4.24E-06
*cd9a*	ENSDARG00000005842	CD9 antigen	37.62	19.91	−2.34	5.21E-05
*eif4a1b*	ENSDARG00000003032	CD68 antigen variant	472.12	258.97	−2.33	1.94E-05
*cd151l*	ENSDARG00000068629	CD151 antigen, like	0.71	4.88	4.80	1.16E-03
*psme3*	ENSDARG00000012234	Proteasome activator complex subunit 3	8.68	19.69	2.02	6.74E-03
*itm1*	ENSDARG00000053832	Integral membrane protein 1(STT3)	121.55	311.95	2.03	6.23E-04
*psme2*	ENSDARG00000033144	Proteasome activator PA28 subunit 2	10.22	25.23	2.04	1.27E-03
*pomp*	ENSDARG00000032296	Proteasome maturation protein	12.60	31.84	2.13	2.01E-03
*tapbpl*	ENSDARG00000058351	TAP binding protein-like(TAPBPL)	5.75	15.38	2.18	1.09E-03
*psme1*	ENSDARG00000002165	Proteasome activator PA28 subunit 1	12.99	39.58	2.65	4.72E-05
*canx*	ENSDARG00000037488	Calnexin	49.15	167.03	2.85	3.71E-06
*cd82b*	ENSDARG00000026070	CD82 antigen,b	5.33	19.59	2.99	5.56E-07
*si:ch211-287j19.6*	ENSDARG00000001470	MHC class I ZE protein	4.96	18.39	3.09	1.63E-06
*itfg1*	ENSDARG00000075584	T-cell immunomodulatory protein	1.74	7.16	3.21	6.74E-07
*hspa4a*	ENSDARG00000004754	Heat shock protein 4a	5.42	18.17	3.28	9.21E-06
*psma5*	ENSDARG00000003526	Proteasome (prosome, macropain) subunit, alpha	1.90	7.52	3.60	9.48E-04
*calr*	ENSDARG00000043276	ER-resident chaperone calreticulin	29.97	131.43	3.72	4.47E-10
*psmb3*	ENSDARG00000013938	Proteasome (prosome, macropain) subunit, beta	13.34	60.15	3.80	3.27E-09
*psmf1*	ENSDARG00000022652	Proteasome (prosome, macropain) inhibitor	3.38	15.05	3.94	1.51E-06
*psmg4*	ENSDARG00000090191	Proteasome (prosome, macropain)	2.39	11.31	4.22	6.25E-04
*hsp90a.2*	ENSDARG00000024746	Heat shock protein HSP 90 kDa alpha 2	0.54	2.81	4.39	7.07E-05
*psmc1b*	ENSDARG00000043561	Proteasome (macropain) 26 S subunit	12.62	67.60	4.55	2.86E-15
*hsp70*	ENSDARG00000021924	Heat shock cognate 70 kDa protein	0.24	2.13	6.67	1.06E-05
*cd63*	ENSDARG00000025147	CD63 antigen	32.31	296.86	7.61	4.69E-20
*cd276*	ENSDARG00000003061	CD276 molecule	0.60	7.15	7.94	2.91E-08
*cd97*	ENSDARG00000089904	CD97 molecule	1.40	13.32	8.03	1.35E-31
*hsp90b1*	ENSDARG00000003570	Endoplasmin (grp94)	93.60	1118.80	10.06	2.85E-18
*gata2a*	ENSDARG00000059327	GATA-binding protein 2a	0.08	1.04	12.76	8.73E-03
*ctsl1a*	ENSDARG00000007836	Cathepsin L, 1 a	20.92	340.42	13.53	1.98E-43
*dnajb11*	ENSDARG00000015088	DnaJ (Hsp40) homolog, subfamily B	3.42	61.99	15.13	1.19E-32

Identification of all differentially expressed genes was based on *p*<0.05. A *p*-value<0.05 indicated that the gene was significantly altered in WED-immunized fish with respect to mock-immunized fish. The absolute value of “Fold-change” is the magnitude of up- or down-regulation for each gene/homolog after WED immunization. Fold-change > 2 indicates up-regulation, and < −2 indicates down-regulation.

Up-regulated genes with established roles in immune responses comprised another large functional category, indicating that active immune-surveillance, immune signaling, and immune cell activation were triggered in the WED-immunized zebrafish liver, like the MHC-I ZE protein (by 3.09-fold). However, the MHC class II DAB, MHC class II beta chain, MHC class II invariant chain (CD74 molecule), MHC class II transactivator (CIITA), and cathepsin S were down-regulated at this stage (Table 3).

**Figure 4:**
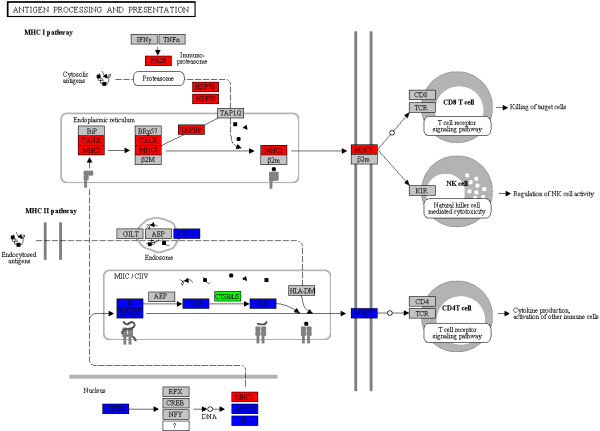
**Significantly differentially expressed genes identified by KEGG as involved in antigen processing and presentation.** Red: significantly increased expression (fold-change >2); Blue: significantly decreased expression (fold-change <0.5); Green: genes detected both in up- and down-regulated groups; Gray: unchanged expression.

To further explore the immune response profiles induced by WED immunization to the level of a single pathway, we used the KAAS web-based pathway analysis program. KEGG analysis was performed to identify genes involved in phagosome and antigen processing and presentation pathways (Figure 4). In the phagosome pathway, 35 genes were identified as strongly up-regulated upon WED immunization, while 15 genes were strongly down-regulated (data not shown). In the antigen processing and presentation signaling pathway, most of the up-regulated genes were found to be interrelated with the MHC-I processing pathway, while most of the down-regulated genes were related to the MHC-II processing pathway. In mammals, antigen processing and presentation are essential for triggering the downstream cellular and/or humoral immune responses [41]. The KEGG results revealed that eight genes involved in the MHC-I pathway were up-regulated, and six genes involved in the MHC-II pathway were down-regulated by two days after WED immunization (Figure 4). These results suggested that the MHC-I related pathways were co-induced following WED immunization, while the MHC-II related pathways were co-depressed. This unique perspective should be further clarified.

### qPCR analysis of MHC processing pathways

**Figure 5:**
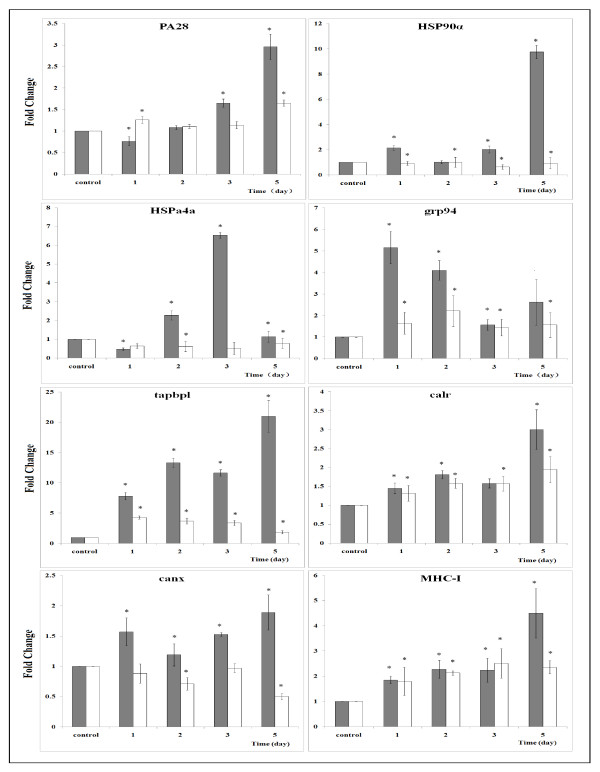
**qPCR analysis of the genes related to MHC-I processing pathway in zebrafish following WED*-*immunization during the first five days.** Proteasome activator (PA28), heat shock protein 90 kDa alpha (HSP90α), heat shock protein 4a (HSPa4a), endoplasmin (grp94), TAP binding protein (tapbpl), calreticulin (calr), calnexin (canx) and MHC class I ZE protein (MHC-I) were validated. The relative expression of the above immune-related genes in livers (gray bars) and spleens (white bars) of zebrafish were analyzed by qPCR. Zebrafish were vaccinated via intramuscular injection with WED or PBS. The livers and spleens of 10 fish were taken at 1, 2, 3, 5 d post-vaccination, respectively, and total RNA was extracted and used for qPCR. The mRNA level of each immune-related gene was normalized to that of β-actin. For the gene of each time point, values represent fold change in expression compared to the control treatment, which was set at 1.0. Results are expressed as means ± SD (n = 3). Mock immunized group was subtracted from each group. Independent-sample *t*-test in the SPSS software (Version 11.5, SPSS Inc.) was used to determine statistical significance of the WED immunized groups relative to mock groups. Significant differences were considered at * *p* < 0.01.

**Figure 6:**
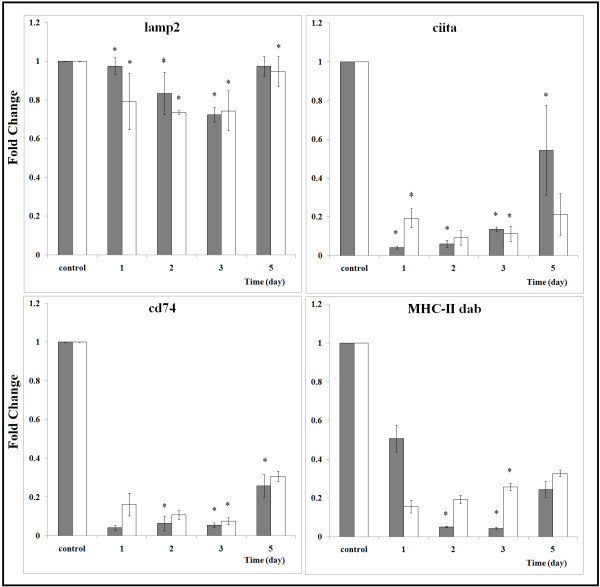
**qPCR analysis of the genes related to MHC-II processing pathway in zebrafish following WED*-*immunization during the first five days.** Lysosomal membrane glycoprotein 2 (lamp2), MHC class II DAB gene (MHC-II dab), CD74 molecule (cd74) and MHC class II, transactivator (CIITA) were validated. qPCR analysis for the expressions of the above immune-related genes in livers (gray bars) and spleens (white bars) of zebrafish vaccinated with WED. The experimental procedure was the same as in the legend of Figure 5.

We next sought to further clarify the strength of the correlations of up-regulated genes to MHC-I processing pathway and down-regulated genes to MHC-II processing pathway in zebrafish during early stage following WED immunization. Differential expression of 12 genes associated with MHC antigen processing was analyzed by qPCR to confirm the hypothesis that antigen processing and presentation pathways elicit an adaptive immune response following immunization. The assay was performed with both spleen and liver samples collected over the first five days post immunization. Most of the results were consistent with those of the RNA-seq analysis (Figure 5 and 6). MHC-I processing pathway related gene expression in liver tissues from WED-immunized groups were significantly up-regulated relative to mock-immunized groups (Figure 5). The up-regulations of differentially expressed genes in liver mostly reappeared in spleen, except for *hsp90α*, *hspa4a* and calnexin. The down-regulation expression of the three genes in spleen might reflect their different functions in two immune-associated organs. In contrast, the MHC-II processing pathway related gene expression was all down-regulated and completely coordinated in liver and spleen during the early stage following WED immunization (Figure 6). This showed that MHC-II processing pathway was inhibited in two immune organs by WED immunization. Thus, CD4+ T cells activation could be depressed following immunization. The implications of this finding should be further investigated in teleost, specifically in zebrafish as model of *E. tarda* infection. In addition, the qPCR data also revealed that antigen processing in liver possesses a comparatively dominant role to that in spleen. The relatively intense expression in liver showed that antigen processing plays an essential role in WED-immunized zebrafish liver.

## Discussion

At present, molecular studies on the immune response to pathogens in fish models are mainly focused on infectious disease pathogenesis. RNA-seq and microarray-based transcriptome profiling studies have revealed that the teleosts are useful *in vivo* models for identifying host determinants of responses to bacterial infection [27-30]. Furthermore, the RNA-seq approach has already been successfully applied to several infectious disease models of zebrafish [25-27]. However, none have applied the RNA-seq technology to elucidate the immune-related pathways underlying the zebrafish response to vaccination for more effective vaccine evaluation. In this work, in order to gain comprehensive insight into the immunogenetics of zebrafish following immunization with the putative *E. tarda* live attenuated vaccine, a high-throughput deep sequencing-by-synthesis technology was used to investigate the immunization-related gene expression patterns. DESeq analysis identified 4565 significantly differentially expressed genes in the zebrafish liver following WED immunization. GO and KEGG analysis revealed that the genes involved in the ER protein processing as well as the phagosome and antigen processing and presentation pathways are regulated at the early stage following WED immunization (Table 1 and Figure 2). Significantly, two class MHC pathways were found to be reversely regulated upon immunization, and the MHC class I pathway was activated and the MHC class II pathway was inhibited (Figures 4,5 and 6). Both the RNA-seq results and qPCR data from our study of zebrafish liver during the early stage after WED immunization indicated that activation of the MHC-I processing pathway in teleosts could elicit cellular immune responses for protection.

Once bacterial vaccines are administrated into the animal host, they are often internalized by phagocytes via different entry mechanism. However, the subsequent issues involved in microbial sensing and antigen processing are not well defined. In the conventional paradigm, MHC class II molecules present antigenic fragments acquired by the endocytic route to the immune system for recognition and activation of CD4+ T cells [42]. MHC class I molecules, on the other hand, are restricted to surveying the cytosol for endogenous antigen from intracellular pathogens (such as bacteria, parasites, and viruses), tumors, or self-proteins, which are degraded into proteasomal products and then presented on MHC class I molecules to CD8+ T cells, thus exersting an irreplaceable role on cellular-mediated immuno-protection toward intracellular pathogens [43,44]. *E. tarda* is believed to be an intracellular pathogen that can survive and replicate within large phagosomes in macrophages [45]. Since WED is an attenuated strain from wild type *E. tarda*, it could be assumed that WED bacteria possess the ability to survive in phagosomes of APC cells and the internalized bacteria are recognized as endogenous or exogenous antigen which would be presented or cross-presented by the MHC-I pathway, and finally evoking a CD8+ CTL-mediated response to achieve immune protection.

In MHC-I antigen processing pathway, antigenic peptides are degraded in the cytoplasm by proteasome, then translocated into the ER and loaded onto MHC-I molecules with the help of several protein components. PA28, as an important proteasome activator, is a heterohexameric ring that binds to one or both ends of the 20 S proteasome [43,46]. Upon binding, it increases the catalytic activity of all three of the proteasome active sites, leads to changes in substrate cleavage, thereby generating more MHC class I-presented peptides [46,47]. Khan *et al.*[48] reported that constitutive proteasomes were replaced with immune-proteasomes in mice livers starting at two days after *Listeria monocytogenes* infection. Immuno-proteasomes support the generation of MHC class I epitopes and shape immune-dominance hierarchies of CD8+ T cells [46]. In mice, this switch is marked by the up-regulation of proteasome activator PA28 subunits, which alter the fragmentation of polypeptides through the proteasome and are inducible by IFN-γ [48]. The study of immune responses to *E. ictaluri* infection in blue catfish liver demonstrated that both the PA28α and PA28β were up-regulated [34]. In the study described herein, the genes encoding PA28 subunit 1, PA28 subunit 2 and PA28 subunit 3 were all up-regulated in zebrafish liver, which suggested a shift toward MHC class I antigen processing occurred at the early stage after WED immunization.

Heat shock proteins (HSP) are a type of highly conserved and ubiquitously expressed proteins that play an essential role as molecular chaperones in protein folding and transport within the cell [49] and possess the ability to stimulate MHC class I antigen processing [50]. HSP/peptide complexes are taken up by APC via specific receptors, whose signaling leads to MHC-I presentation of HSP-associated peptides and the induction of specific CD8+ cytotoxic T cells [50]. The antigenic peptides chaperoned by HSPs are known to be more efficient, by orders of magnitude, than the free peptides for presentation by MHC-I [49,50]. In our work, three heat shock proteins (heat shock cognate 70 kDa protein, heat shock protein 4a and heat shock protein 90 kDa alpha 2) were found to be up-regulated following WED immunization, and the activated HSPs suggested that the internalized WED bacteria were processed and loaded onto MHC class I molecules, ultimately initiating initiate the CTLs.

As cited above, MHC class I molecules present antigenic peptides on cell surface for recognition by CD8+ T cells [43]. Like other glycoproteins, the folding and assembly of MHC class I molecules require interactions with a number of chaperone molecules in the ER, some of which are specific to MHC class I molecules [44]. Among the known ER chaperones, endoplasmin (grp94) possesses the ability to bind peptides suitable for assembly on to MHC class I molecules together with calreticulin [51]. Calreticulin and calnexin are specialized ER lectin-binding chaperones to bind transiently to newly-synthesized glycoproteins, but the calreticulin has been suggested as unique to interactions with the HSP/grp94 complex, which leads to recruitment of ER protein 57 [52]. The interaction between calnexin and MHC class I molecules is believed to stabilize the class I heavy chain and help it to associate with the β2m component [51,53]. In this work, the three ER chaperons, calreticulin, calnexin and endoplasmin (grp94), were all found to be induced in WED-immunized zebrafish liver, providing further evidence that an active MHC class I processing pathway was stimulated by WED immunization. In addition, TAP binding protein, another molecule involved in MHC class I antigen loading [44,49,51,53], and MHC class I complex ZE protein were also up-regulated in WED-immunized zebrafish liver, strongly suggesting a vigorous activation of the MHC-I processing pathway.

The MHC antigen processing-associated genes from zebrafish have been extensively characterized. However, little is known about their expression patterns in zebrafish following vaccine immunization. Recently, the coordinated up-regulation of MHC class I-related components including MHC class I alpha chain, β2m, calreticulin, endoplasmin, PA28α and PA28β were reported in large yellow croaker following poly I:C injection [54] and in catfish following an intracellular bacterial infection [34]. In this work, the RNA-seq data were given to show a coordinated down-regulation of several MHC class II antigen processing and presentation components, including the MHC-II DAB, MHC-II beta chain, MHC-II invariant chain (CD74), MHC class II transactivator (CIITA), cathepsin B and lysosomal membrane glycoprotein 2 (lamp2). This complex process is illustrated in Figure 4 and the differentially expressed genes are listed in Table 3. Furthermore, qPCR data confirmed the co-inhibition of lamp2, MHC-II dab, CD74, and CIITA in zebrafish liver and spleen (Figure 6). In previous researches, a remarkable inhibition of MHC-II expression and antigen presentation was ever reported in some pathogen infection models, including *Brucella abortus*[55], and *Mycobacterium tuberculosis*[56-58]. For pathogens, an ability to impair the antigen processing and presentation of host has been proposed to facilitate chronic infection by decreasing T cell responses to microbial antigens. For vaccines, however, the underlying significance of suppression of the MHC-II expression and antigen presentation remains unknown.

## Conclusions

In conclusion, in this work, zebrafish was used as a model to investigate the host immune mechanisms underlying the protective effects of the *E. tarda* live attenuated vaccine. RNA-seq data revealed that the coordinate up-regulation of MHC-I processing pathways and down-regulation of MHC-II-associated pathways occurred at the early stage of vaccine immunization, providing insights into the molecular mechanisms of immune protection. The successful application of RNA-seq technology in the vaccine-zebrafish interaction model in this work established a new experimental platform for investigating the vaccine-specific host immune responses in a comprehensive and sensitive manner. Future studies using this approach will likely provide further significant insights into the detailed mechanisms of teleost immunity that will benefit the aquaculture industry, both from economic and human food source perspectives.

## Methods

### Fish and immunization

Healthy zebrafish (*Danio rerio*), weighing 0.3 ± 0.1 g and about 6 months of age, were obtained from the animal center at the East China University of Science and Technology (Shanghai, China) and maintained at 22 ± 2°C in a zebrafish cultivation system with a photo-period of 12:12 h (light : dark). Aquaria were supplied with flow-through dechlorinated and continuously aerated water at a rate of approximately 2×10^-4^ min^-1^. After at least one week of acclimatization, they were randomly divided into six treatment groups (70 fish per group) including three immunized groups (V1-V3) and three control groups (C1-C3), and the fish in each group were cultured in a separate tank. The fish in V1-V3 groups were intramuscularly (i.m.) injected with 1×10^5^ CFU.fish^-1^ of WED bacteria in 5 μl phosphate buffered saline (PBS), as previously described [12], and the fish in C1-C3 groups were i.m. injected with 5 μl PBS alone. After two days of immunization, 20 fish from each of the three WED-immunized and three mock-immunized groups were sacrificed under anesthesia to obtain liver samples, and subsequently stored at −80°C until RNA extraction for RNA-seq analysis. Meanwhile, 10 fish from each group were sacrificed under anesthesia at days 1, 2, 3, and 5 post-immunization to obtain liver and spleen tissue samples, and subsequently stored at −80°C until RNA extraction for real-time qPCR analysis. All the zebrafish were handled in compliance with the local animal welfare regulations and maintained according to standard protocols (http://ZFIN.org). The immunization experiment was approved by the animal center at the East China University of Science and Technology (Shanghai, China).

### Library preparation and sequencing

Total RNA was extracted from each tissue sample using the TRIzol reagent (Invitrogen, USA) according to the manufacturer’s instructions. To remove residual genomic DNA, the RNA samples were incubated with 10 units of DNA-free DNAse I (Ambion, USA) for 30 min at 37°C. The quality and quantity of the purified RNA were determined by measuring the absorbance at 260 nm/280 nm (A_260_/A_280_) using a Nanodrop ND-1000 spectrophotometer (LabTech, USA). RNA integrity was further verified by electrophoresis through a 1.5% (w/v) agarose gel.

Poly (A) mRNA was isolated from the total RNA samples with oligo (dT) magnetic beads (Invitrogen). The purified mRNA was fragmented by the RNA fragmentation kit (Ambion) and applied as template for first-strand cDNA synthesis using random hexamer primers and reverse transcriptase (Invitrogen). The second-strand cDNA was synthesized using RNase H (Invitrogen) and DNA polymerase I (New England Biolabs, USA). The Illumina Genomic DNA Sample Prep kit (Illumina, USA) was used to generate 120 bp paired-end (PE) cDNA libraries by following the manufacturer’s protocol. The libraries were loaded onto flow cell channels for sequencing on the Illumina HiSeq 2000 instrument by the Chinese National Human Genome Center (Shanghai, China). A total of six paired-end cDNA libraries of zebrafish livers were constructed for each of the test groups of WED-immunized and mock-immunized fish. Triplicate biological replicates were performed for each group. Raw data (tag sequences) were deposited in the NCBI database under submission number SRA048658.2.

### Transcriptome analysis

The Illumina HiSeq 2000 system-generated 120 bp raw PE reads were first processed by the FASTX-Toolkit to remove the reads with sequencing adaptors and of low quality (phred quality <5). Then, the Burrows-Wheeler Aligner’s Smith-Waterman Alignment (BWA) program was used to align the remaining reads to the reference zebrafish mRNA from the Ensembl database [59]. The transcription level of each gene was deduced by determining the total number of reads mapped to each gene using Picard tools (http://picard.sourceforge.net/). Differentially expressed genes were identified by the DESeq package in R software [60], using two-fold change (log_2_ (fold-change) ≥ 1 or ≤ -1) and *p*-value<0.05 (cut-off at 5% false discovery rate (FDR)) as the threshold. After data normalization by the *p*-value and FDR calculation, the resulting expression intensity values were analyzed by the MA plot-based method, as described by Wang *et al.*[32].

### Functional analysis of differentially expressed genes

The Database for Annotation, Visualization and Integrated Discovery (DAVID, v6.7) [61] was used to investigate functional enrichment for over- and under-expressed genes by more than two-fold in the WED-immunized group relative to the mock-immunized group. Gene functional enrichment was performed using the default parameters in DAVID to obtain an adjusted *p*-value <0.05 for the test gene group versus the zebrafish gene ontology (GO) annotation set. The fold-enrichment cut-off suggested for DAVID functional annotation is 1.5 [61]. In addition, the significantly up-regulated genes from the differentially expressed genes dataset were further analyzed by investigating the corresponding GO biological processes. Furthermore, GO analysis of genes transcribed at different levels was also performed using the Biological Networks Gene Ontology (BiNGO) tool, which is based on the Cytoscape software (http://www.cytoscape.org). The hypergeometric test with Benjamini & Hochberg False Discovery Rate (FDR) was performed using the default parameters to obtain an adjusted *p*-value (<0.05) between the test gene group and the merged non-redundancy zebrafish (*Danio rerio*) and mouse (*Mus musculus*) GO annotation set. Finally, the web-based Kyoto Encyclopedia of Genes and Genomes (KEGG) pathway analysis program run by the KEGG Automatic Annotation Server (KAAS) (http://www.genome.jp/tools/kaas/) was used to obtain functional annotation of genes by performing basic local alignment search tool (BLAST)-mediated comparisons against the manually-curated KEGG GENES database [62]. We merged the most current KEGG GENES entries for *Danio rerio* and *Mus musculus* to generate a reference dataset and used the bi-directional best hit (BBH) information method to further analyze the significantly differentially expressed genes to gain insights into the related biological pathways.

### qPCR analysis

To verify the differential expression detected by sequencing, qPCR was performed using the ABI Prism 7500 Detection System (Applied Biosystems, USA) with SYBR Green (Roche, USA) as the fluorescent detection dye, according to the manufacturer’s protocol. First-strand cDNA was synthesized from 1 μg of total mRNA, as described above, and applied as a template for qPCR with gene-specific primers (Additional file 4). Primers were designed using Primer Express 3 software. To determine the PCR efficiency, we first generated a standard curve by amplifying ten-fold serial dilutions of cDNA using primers to both the gene of interest and an internal control (β-actin), and all primers were optimized until PCR efficiency values fell in 1.80-2.15. The qPCR thermal cycling conditions for all reactions were 95°C for 15 min, followed by 40 cycles of 95°C for 5 s, 60°C for 20 s, and 72°C for 20 s. All qPCR reactions were performed for three biological replicates, and the data for each sample were expressed relative to the expression levels of β-actin by using the 2^-ΔΔCT^ method [63]. Independent-sample *t*-test in the SPSS software (Version 11.5, SPSS Inc.) was used to determine statistical significance. Significant differences were considered at *p*<0.01.

## Supplementary Material

Additional file 1: **Solexa libraries of the WED immunized and PBS mock zebrafish liver.** Six Solexa cDNA libraries were constructed from the livers of mock-immunized and WED-immunized zebrafish. Biological replicates (C1-C3 and V1-V3) were pooled to make representative samples for deep sequencing analysis. To assess the quality of sequencing, the reads were mapped to the zebrafish reference genome with no more than 5-mismatches. The sequencing data for C1-read 1 to C3-read 2 and V1-read 1 to V3-read 2 corresponded to the paired end sequencing (forward and reverse sequencing) data in each library, respectively. Click here for file

Additional file 2: **Annotation results of 4565 differentially expressed genes.** Transcription level of each gene was deduced by determining the total number of reads mapped to each gene using Picard tools. Differentially expressed genes were identified by the DESeq package in R software using two-fold change (log_2_ (fold-change) ≥1 or ≤-1) and *p*-value <0.05 (cut-off at 5% false discovery rate (FDR)) as the threshold. “baseMean”, the base mean of counts divided by the size factors. “baseMean A”, the base mean for the counts of PBS-mock condition. “baseMean B”, the base mean for the counts of WED-immunized condition. “rpkm_avg_c”, the expression level of PBS-mock group. “rpkm_avg_v”, the expression level of WED-immunized group. “ZF-Annotation”, annotation of the differentially expressed genes through BLASTN similarity searches against the Ensembl zebrafish RefSeq mRNA database. Click here for file

Additional file 3: **GO function annotation results of 4565 differentially expressed genes.** Functional enrichment for over- and under-expressed genes by more than two-fold in the WED-immunized group relative to the mock-immunized group generated by DAVID (v6.7). Gene functional enrichment was performed using the default parameters in DAVID to obtain an adjusted *p*-value <0.05 for the test gene group versus the zebrafish gene ontology (GO) annotation set. The fold-enrichment cut-off suggested by DAVID functional annotation was 1.5. The Biological Progress (BP-up and BP-down), Cellular Components (CC-up and CC-down), and Molecular Functions (MF-up and MF-down) associated with the GO analysis at different levels were also analyzed by BiNGO software. The hypergeometric test with Benjamini & Hochberg FDR were performed by the default parameters to obtain an adjusted *p*-value (<0.05) between the test gene group and the merged non-redundancy zebrafish (*Danio rerio*) and mouse (*Mus musculus*) GO annotation set. Click here for file

Additional file 4: Primers for quantitative real-time PCR. Click here for file
