# Supplementary material for: RNA-seq liver transcriptome analysis reveals an activated MHC-I pathway and an inhibited MHC-II pathway at the early stage of vaccine immunization in zebrafish
Source: BMC Genomics. 2012 Jul 17;13:319. doi: 10.1186/1471-2164-13-319 (PMC3583171; doi:10.1186/1471-2164-13-319)
Supplement: Additional file 4: — Primers for quantitative real-time PCR. [file 1471-2164-13-319-S4.docx]

**Additional file 4. Primers for quantitative real-time PCR**

| **Gene name** | **Forward primer (5′-3′)** | **Reverse primer (5′-3′)** |
| --- | --- | --- |
| *β-actin* | ATGGATGAGGAAATCGCTGCC | CTCCCTGATGTCTGGGTCGTC |
| *MHC-I* | GGCTGTTTTTGCCGCTCTG | GTGGACAGGTCTGGATAAAG |
| [*MHC-IIdab*](http://zfin.org/action/marker/view/ZDB-GENE-980526-200) | CTCTGTGGGGAAGTTTGTG | CCAGATCCGAGCATTATGTC |
| [*cd74*](http://zfin.org/action/marker/view/ZDB-GENE-990910-10) | GGGACCTCAGCCAAGAAATAAG | CTCCTCGTCTCTCCAGGGTGTTG |
| [*grp94*](http://zfin.org/action/marker/view/ZDB-GENE-031002-1) | TGACGAGGTTGTTCAGAGGGAG | CAAGGCATCGGAAGCATTAGAG |
| *tapbpl* | ACAGAGGTCTTCATCAGG | GTGTTTGTTGCTCCACTTTG |
| [*hspa4a*](http://zfin.org/action/marker/view/ZDB-GENE-040426-2832) | TGCTGATGTGGTTCTGTCCTGCTC | ACCTTCATTCTCAACAAAATCCCTG |
| *PA28* | TGGGATAAGACATTACACG | GTCTAAGGAAGTCATTGCTGC |
| [*canx*](http://zfin.org/action/marker/view/ZDB-GENE-060929-708) | ACAGTTAACCGACACATCGCCAATA | CTTCCTCTTCATCCTCCAGGTCTTC |
| [*calr*](http://asia.ensembl.org/Danio_rerio/Gene/Summary?g=ENSDARG00000043276) | GCCAATGGAAACTGACCTC | CTTCTGCTCGTGTTTTACCG |
| *ciita* | GTCCCTCCTGGATGGTTCG | TAGATGTTTTTTCTGGTATGC |
| *hsp90a* | ACTAGAACCCGCTGACGC | CTCAGCCTGAAACGCAAAC |
| *lamp2* | TATCCTGCTGAGTGGTATTG | GTAGATGAAGTAGTCTGGATG |
